# A Study of 41 Canine Orthologues of Human Genes Involved in Monogenic Obesity Reveals Marker in the *ADCY3* for Body Weight in Labrador Retrievers

**DOI:** 10.3390/vetsci10060390

**Published:** 2023-06-08

**Authors:** Mateusz Sypniewski, Maciej Szydlowski

**Affiliations:** Department of Genetics and Animal Breeding, Poznan University of Life Sciences, Wołyńska 33, 60-637 Poznań, Poland

**Keywords:** GWAS, linear mixed models, obesity, dogs, whole genome sequencing

## Abstract

**Simple Summary:**

Obesity is a prevalent problem in dogs, although individual susceptibility is determined by a combination of genetic and non-genetic risk factors. This study looked at 41 canine orthologues of human genes linked to obesity in humans to find genes linked to weight in Labrador Retriever dogs. A linear mixed model using sex, age, and sterilization as covariates and population structure as a random effect were used to evaluate 11,520 variants in 50 adult dogs. A statistically significant variant in the *ADCY3* gene was discovered throughout the study, making it a promising marker for canine obesity research. Our findings provide further evidence that the genetic makeup of obesity in Labrador Retriever dogs contains genes with large effect sizes.

**Abstract:**

Obesity and overweight are common conditions in dogs, but individual susceptibility varies with numerous risk factors, including diet, age, sterilization, and gender. In addition to environmental and biological factors, genetic and epigenetic risk factors can influence predisposition to canine obesity, however, they remain unknown. Labrador Retrievers are one of the breeds that are prone to obesity. The purpose of this study was to analyse 41 canine orthologues of human genes linked to monogenic obesity in humans to identify genes associated with body weight in Labrador Retriever dogs. We analysed 11,520 variants from 50 dogs using a linear mixed model with sex, age, and sterilization as covariates and population structure as a random effect. Estimates obtained from the model were subjected to a maxT permutation procedure to adjust *p*-values for FWER < 0.05. Only the *ADCY3* gene showed statistically significant association: TA>T deletion located at 17:19,222,459 in 1/20 intron (per allele effect of 5.56 kg, SE 0.018, *p*-value = 5.83 × 10^−5^, TA/TA: 11 dogs; TA/T: 32 dogs; T/T: 7 dogs). Mutations in the *ADCY3* gene have already been associated with obesity in mice and humans, making it a promising marker for canine obesity research. Our results provide further evidence that the genetic makeup of obesity in Labrador Retriever dogs contains genes with large effect sizes.

## 1. Introduction

Obesity is the most common type of malnutrition in dogs [1,2,3,4,5,6]. Individual susceptibility arises from interaction between environmental (e.g., diet and physical activity), biological (age, sex or sterilization), genetic (including breed), and epigenetic risk factors [7]. In humans, the cause of obesity may be monogenic or polygenic. Monogenic obesity is characterized by the occurrence of single mutations affecting the functions of entire genes and metabolic pathways. In the case of common polygenic obesity, the inheritance mechanism typical for polygenic diseases is observed, where the accumulation of many mutations and non-genetic factors contributes to the phenotype. Despite the distinction of these two categories, the occurrence of monogenic obesity is to some extent conditioned by polygenic predispositions, and genes linked to human monogenetic obesity are important for polygenic obesity [8].

The Labrador Retriever breed is a popular dog breed in many countries. Regardless of location, Labrador populations are at risk of becoming obese and overweight. Multivariate analysis adjusted for geographic region, diet, and age found that out of 1738 tested Labrador Retrievers, 35.9% dogs were classified overweight and 5.2% obese [9]. This suggests an established predisposition for this breed to develop obesity in adulthood. At the same time, dietary regime and exercise allow dogs to maintain the correct weight, which is typical of polygenic obesity. The polygenic nature of obesity does not exclude the participation of genes with a high influence that contribute to individual genetic variability within the breed [10]. Knowledge of genes with large effects could be useful for breeders to limit risk variants in populations. So far, knowledge of the individual effects of obesity genes is very limited and is mainly based on candidate gene studies [10,11,12]. 

It is expected that polygenes can explain a significant portion of intra-breed phenotypic variation in obesity, however, reliable estimates of heritability are missing. Sequencing of candidate genes for obesity in Labrador Retriever dogs identified a 14 bp deletion in the *POMC* gene that has a large effect on body weight, a trait strongly correlated with obesity [10,13]. This finding not only indicates that the genetic architecture of obesity may include genes with large effects, but also proves that the candidate gene approach can be an effective method of identifying genetic risk factors in small canine populations.

The aim of this study was to analyse 41 canine orthologues of genes linked to human obesity to identify those which have associations with body weight in Labrador Retriever dogs. To minimise risk of false positive results due to limited population size, we used data on whole genome sequencing (WGS) to adjust for linkage disequilibrium (LD) and population structure. Our limited research may provide another source of future meta-analysis.

## 2. Material and Methods

### 2.1. Animals and Phenotypes

We analysed 50 Labrador Retriever dogs (32 females and 18 males) with age ranged between 12 to 120 months. The analysed phenotype was body mass in kilograms and ranged between 25–50 kg with mean of 36.13 (SD 6.43). Pedigree data were not available. The records were collected from 2014 to 2017 in two veterinary clinics during routine visits.

### 2.2. Genotypes

We analysed the canine orthologs of 41 genes associated with monogenic obesity in humans. The list of gene names was taken from a commercial panel offered by the Blueprint Genetics (Monogenic Obesity Panel; test code KI1701; https://blueprintgenetics.com/; accessed on 12 September 2022). The gene names with the location in the canine reference genome (CanFam3.1) are presented in Supplementary Table S1. We decided to add RYR3 gene to the study because our preliminary analyses on structural variants suggested that it may be important for body condition score in Labrador retriever. Information on analysed genes were taken from the human GeneCards database [14] (accessed on 12 September 2022) and mouse genome Database [15] (accessed on 12 September 2022). The whole genome sequences for the fifty dogs were available from previous study [16]. The raw sequence data are publicly available (Nucleotide Archive PRJEB47658). The procedure for genotype calls for SNPs and short variants was as described by Szydlowski and Antkowiak [16]. All variants were annotated with VEP ver. 104 [17]. For the procedures of variant inclusion, we used PLINK V.1.9 software [18]. We included variants with call rate > 95%, minor allele frequency (MAF) > 5%, and in Hardy–Weinberg equilibrium (Fisher exact test *p*-value > 10^−6^). Following the filtering, 11,520 variants remained for association analysis.

### 2.3. Association Analysis

False discoveries in association studies are a major concern which can be attributed to spurious associations caused by population structure and cryptic relatedness between individuals [19,20]. To minimise risk of false positive results, we applied a linear mixed model (LMM), in which genetic similarity between individuals was incorporated as the variance–covariance structure of the random effect for individuals. This method allows adjustment of effect estimates of each marker for population structure and linkage disequilibrium. Despite the advantages of LMM, testing of thousands of markers simultaneously in a small sample would lead to a large number of false positive associations. To further minimize false positives, we estimated significance threshold with the maxT permutation method [21]. In this approach, a maximal test statistic computed over all markers in each of their permutations is taken to estimate significance threshold adjusted for family-wise error rate (FWER) where the FWER is defined as the probability of making at least one type-I error (or false positive). A recent study by John et al. [22] proposed a new approach which combines LMM and maxT permutation with small computational burden, named permGWAS. To test the association between weight and each variant, we applied permutation-based association study with a linear mixed model (LMM) using the permGWAS method. Age, sex, and sterilisation status were fitted in the statistical model as covariates. For selection of statistically significant variants, we applied FWER < 0.05 significance threshold. We used plink 1.9 software for data filtering [18] and permGWAS software for association analysis (https://github.com/grimmlab/permGWAS; accessed on 21 September 2022; [22]). The Manhattan plot was generated using ggplot2 package in R [23,24].

## 3. Results

**Figure 1:**
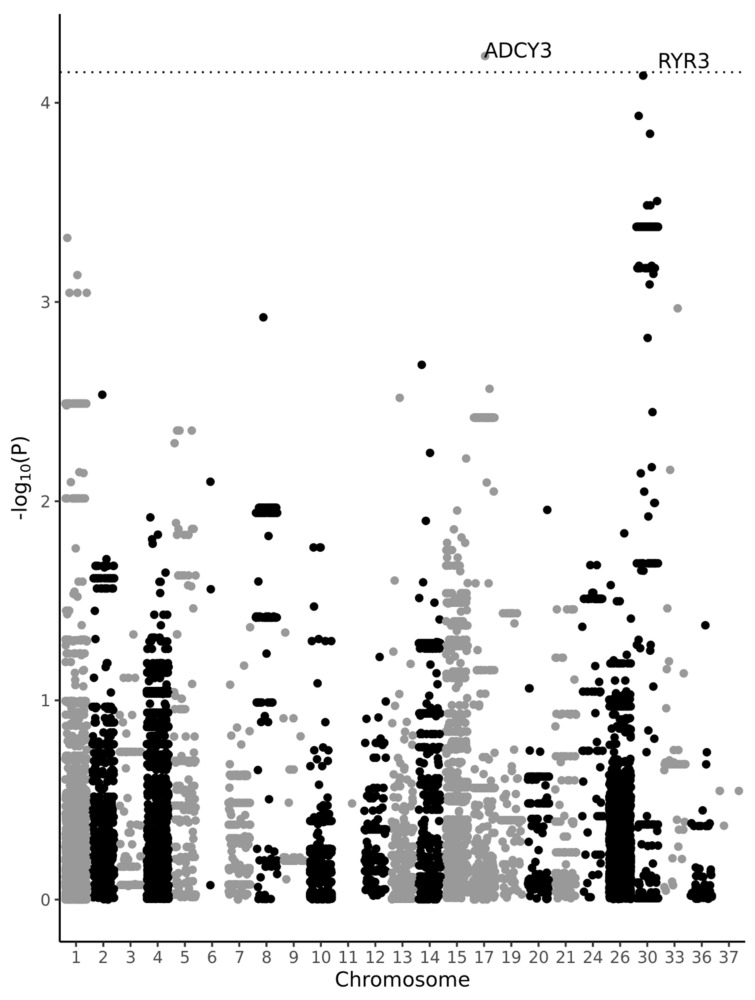
Manhattan plot for the 11,520 variants in the canine genome. Dotted line indicates FWER threshold < 0.05. One position within the *ADCY3* gene occurred with statistical significance (17:19222459 TA>T with *p*-value = 5.83 × 10^−5^).

In total, we analysed 41 genes with 11,520 short variants. The permGWAS took approx. 9 min to estimate FWER adjusted *p*-values with 10,000 permutations. One variant in the *ADCY3* gene passed FWER < 0.05 significance threshold (Figure 1). The variant is a TA>T deletion located at 17:19,222,459 in 1/20 intron (per allele effect of 5.56 kg, SE 0.018, *p*-value = 5.83 × 10^−5^, TA/TA: 11 dogs; TA/T: 32 dogs; T/T: 7 dogs). The second, most significant variant, slightly below significance threshold, was G>A substitution in the *RYR3* gene (30:1,294,982, intron 46/130, per allele effect of 5.97 kg, SE 0.018, *p*-value = 7.31 × 10^−5^, G/G: 25 dogs, G/A: 19 dogs, AA: 6 dogs). 

For *POMC* 14 bp deletion (17:19431807-19431820 CGCGGCGGGGCCCT>C) no significant association was detected.

## 4. Discussion

Out of 41 tested genes, only *ADCY3* yielded a statistically significant result. ADCY3 catalyses the synthesis of cyclic AMP (cAMP) from ATP. Cyclic AMP is an essential second messenger in intracellular signalling downstream of key metabolic mediators such as glucagon-like peptide 1, ghrelin, and α-melanocyte-stimulating hormone [25], and cAMP signalling has been linked to insulin secretion in beta cells [26]. Loss-of-function mutations in this gene are linked to early onset of severe obesity in humans [27,28,29]. In addition, mouse models have indicated that ADCY3 has an important role in the regulation of glucose metabolism and adiposity [26]. Mutations in *ADCY3* in mice cause impaired insulin sensitivity and dyslipidaemia [30] and mice with *ADCY3* knockout show increased fat mass, hyperphagia, depression-like phenotypes, and leptin resistance [31,32]. Furthermore, a common variation in *ADCY5*, a gene in the same family as *ADCY3*, is known to be associated with fasting plasma glucose levels and risk of type 2 diabetes [33]. Studies on mice showed that gain-of-function mutation in *ADCY3* reduced white and brown adipose tissue depots and protected the animals from hepatic lipid accumulation when fed with a high-fat diet [34]. Although the 17:19,222,459 TA>T variant is located in the intron, it has been proven that introns play a role in synthesis of noncoding RNA [35] and regulation of gene expression [36,37]. Moreover, epigenomic study in a human cohort identified rare variants within the 2p23.3 region making this region more susceptible to methylation. The result is lowered expression of *ADCY3* gene which is associated with BMI increase [7]. Unfortunately, to the best of the authors’ knowledge there have been no similar studies for dogs. Furthermore, we cannot rule out that the influence of the *ADCY3* gene observed here is an indirect effect, correlated with a structural mutation that was not investigated in our study.

The second most significant variant was in the *RYR3* gene. Despite not passing the significance threshold, there are some indicators linking this gene with obesity. *RYR3* encodes ryanodine receptor 3, a high-conductance cation channel, which releases calcium ions from intracellular storage [38,39,40,41,42]. Calcium modulators such as RYR3 can regulate adiponectin expression, which is an important adipose-specific protein responsible for, e.g., insulin sensitizing [43]. Study on *RYR3* knockdown nice identified that *RYR3* plays role in regulation of adiponectin expression. Silencing of *RYR3* in high-fat-fed obese mice increased serum adiponectin level, improved INS sensitivity, and lowered fasting glucose levels [44]. 

Previous studies [10,13] reported the effect of 14 bp deletion in the *POMC* gene, however, no significant association was detected in our study. An explanation is the low frequency of this variant in our cohort (12 heterozygous and a single homozygous).

Our research has a number of obvious weaknesses that must be considered if false conclusions are to be avoided. First, despite a number of efforts to reduce false positives in this small population, there is no guarantee that the risk of false positives has been sufficiently reduced. Second, our results may be false not only because of the small sample, but also because of its heterogeneity. Thirdly, the conducted studies do not explain the mechanism of the association observed here, which is particularly important for the mutation located in the intron. The three limitations mentioned here, among other weaknesses of our research, force us to treat the obtained results with caution.

## 5. Conclusions

The findings of this study suggest that intronic mutation in the *ADCY3* gene may be an important statistically linked risk factor for obesity in the Labrador Retriever breed. Mutations in the *ADCY3* gene have already been linked to obesity in mice and humans, making the gene a promising marker for obesity research in dogs. Our results provide further evidence that the genetic architecture of obesity in Labradors Retriever dogs includes genes with large effect sizes. Our report provides another source of data for a meta-analysis aimed at identifying robust genetic risk factors for obesity.

## Data Availability

The raw sequence data is publicly available at Nucleotide Archive PRJEB47658.

## Supplementary Materials

The following supporting information can be downloaded at: https://www.mdpi.com/article/10.3390/vetsci10060390/s1, Table S1: The 41 genes linked to monogenic obesity in humans, and their orthologs in the canine genome.
