# Supplementary material for: A Study of 41 Canine Orthologues of Human Genes Involved in Monogenic Obesity Reveals Marker in the ADCY3 for Body Weight in Labrador Retrievers
Source: Vet Sci. 2023 Jun 8;10(6):390. doi: 10.3390/vetsci10060390 (PMC10301554; doi:10.3390/vetsci10060390)
Supplement: Supplementary file 1 [file vetsci-10-00390-s001.zip › vetsci-2356759-supplementary.pdf]

Table S1. The 41 genes linked to monogenic obesity in humans, and their orthologs in the canine genome.

HUGO gene ID	Ensembl gene ID (CanFam3.1)	Position
MC4R	ENSCAFG00000000090	1: 16131589-16133079
NTRK2	ENSCAFG00000001380	1: 74449060-74777032
DYRK1B	ENSCAFG00000005428	1: 113644100-113828488
NR0B2	ENSCAFG00000012261	2: 73234586-73236206
PAX7	ENSCAFG00000015539	2: 79815748-73236206
PCSK1	ENSCAFG00000007882	3: 13233594-13274094
MAGEL2	ENSCAFG00000010136	3: 36363930-36365870
ABLIM3	ENSCAFG00000018323	4: 59690735-59798383
GHR	ENSCAFG00000018579	4: 67022252-67290473
LEPR	ENSCAFG00000018600	5: 44688109-44775636
CDH15	ENSCAFG00000019846	5: 64258741-64277190
SH2B1	ENSCAFG00000017219	6: 18371832-18380962
SDCCAG8	ENSCAFG00000015803	7: 34396345-34631462
TTC8	ENSCAFG00000017478	8: 60061732-60115075
MKS1	ENSCAFG00000017464	9: 32860995-32879338
WDPCP	ENSCAFG00000003119	10: 62877527-63316345
TRIM32	ENSCAFG00000003516	11: 70462007-70463974
PHIP	ENSCAFG00000002804	12: 39904603-40032194
SIM1	ENSCAFG00000003538	12: 58485823-58553192
VPS13B	ENSCAFG00000000507	13: 1101610-1871225
LEP	ENSCAFG00000001672	14: 8116122-8131395
BBS9	ENSCAFG00000003161	14: 45200458-45643645
BBS10	ENSCAFG00000031800	15: 19331876-19335583
CEP290	ENSCAFG00000006027	15: 29194983-29280429
CPE	ENSCAFG00000008861	15: 61321227-61414789
ADCY3	ENSCAFG00000004090	17: 19154571-19232411
POMC	ENSCAFG00000004149	17: 19431585-19434440
ALMS1	ENSCAFG00000008827	17: 49301606-49513625
BBS12	ENSCAFG00000003998	19: 17538346-17540487
BBS7	ENSCAFG00000004128	19: 18280520-18315825
PPARG	ENSCAFG00000004991	20: 6050357-6207981
UCP3	ENSCAFG00000042759	21: 24277896-24288639
TUB	ENSCAFG00000006892	21: 31566130-31625101
MKKS	ENSCAFG00000005651	24: 11898236-11913740
MC3R	ENSCAFG00000011887	24: 41492371-41494033
GNAS	ENSCAFG00000023756	24: 43643405-43658657
KSR2	ENSCAFG00000009865	26: 13987827-14395111
RYR3	ENSCAFG00000008056	30: 1180359-1533619
ARL6	ENSCAFG00000009111	33: 4800917-4839635
CEP19	ENSCAFG00000013046	33: 29735351-29741128
BBS5	ENSCAFG00000041315	36: 14207014-14233633
MSTN	ENSCAFG00000009398	37: 729172-734362
